# Sexual dimorphism in shells of *Cochlostoma septemspirale* (Caenogastropoda, Cyclophoroidea, Diplommatinidae, Cochlostomatinae)

**DOI:** 10.3897/zookeys.208.2869

**Published:** 2012-07-17

**Authors:** Fabian Reichenbach, Hannes Baur, Eike Neubert

**Affiliations:** 1 University of Bern, Institute of Ecology and Evolution, Baltzerstrasse 6, 3012 Bern, Switzerland; 2 Naturhistorisches Museum der Burgergemeinde Bern, Abteilung Wirbellose Tiere, Bernastrasse 15, 3005 Bern, Switzerland

**Keywords:** Sexual dimorphism, shell features, *C. septemspirale*, PCA ratio spectrum, allometry ratio spectrum, LDA ratio extractor

## Abstract

Sexual dimorphisms in shell-bearing snails expressed by characteristic traits of their respective shells would offer the possibility for a lot of studies about gender distribution in populations, species, etc. In this study, the seven main shell characters of the snail *Cochlostoma septemspirale* were measured in both sexes: (1) height and (2) width of the shell, (3) height and (4) width of the aperture, (5) width of the last whorl, (6) rib density on the last whorl, and (7) intensity of the reddish or brown pigments forming three bands over the shell. The variation of size and shape was explored with statistical methods adapted to principal components analysis (PCA) and linear discriminant analysis (LDA). In particular, we applied some multivariate morphometric tools for the analysis of ratios that have been developed only recently, that is, the PCA ratio spectrum, allometry ratio spectrum, and LDA ratio extractor. The overall separation of the two sexes was tested with LDA cross validation.

The results show that there is a sexual dimorphism in the size and shape of shells. Females are more slender than males and are characterised by larger size, a slightly reduced aperture height but larger shell height and whorl width. Therefore they have a considerable larger shell volume (about one fifth) in the part above the aperture. Furthermore, the last whorl of females is slightly less strongly pigmented and mean rib density slightly higher. All characters overlap quite considerably between sexes. However, by using cross validation based on the 5 continuous shell characters more than 90% of the shells can be correctly assigned to each sex.

## Introduction

Molluscs can be hermaphrodites or gonochoristic organisms with separate sexes throughout their life span, with the latter group sometimes being subject to differing phenotypic traits. Sexual dimophism has been recorded for unionoid mussels as well as for some marine, freshwater and terrestrial snails (for examples see: Raven 1990; Kenchington and Glass 1998; Gofas 2001; Minton and Wang 2011). In gastropods there are two large groups, the hermaphroditic pulmonate snails, and the remaining groups (formerly called prosobranch molluscs) with separate sexes, bearing an operculum to close the shell. The latter group mainly inhabits marine habitats, while the former group forms the majority of terrestrial species. However, there are several “prosobranch” families, which successfully adapted to terrestrial life. As in all other bisexual organisms, sexual dimorphism may occur, and thus there is the question whether this phenomenon can be found here as well.

Sex determination without harming the living individuals often is a problem. Usually, the animals hide inside the shell, what makes it impossible to determine its sex. The solution for this problem could be a method that helps to distinguish between the sexes based on sex-related shell features. This could also help to analyse the sex ratio within given populations even when only shells are available, as often is the case in malacology and particularly in museum’s collections.

Quite recently, De Mattia et al. (2011) intensively discussed the *Cochlostoma* species from Sabotino Hill (NE Italy), where three species of this genus occur. Next to anatomical and conchological character states, the authors also used landmark based, geometric morphometry to obtain reliable shell discriminating characters. To avoid a bias caused by sexual dimorphism, De Mattia et al. (2011) used three male and female specimens of each species, but the contribution of each sex to the average shell form was not addressed by them.

**Figure 1.**
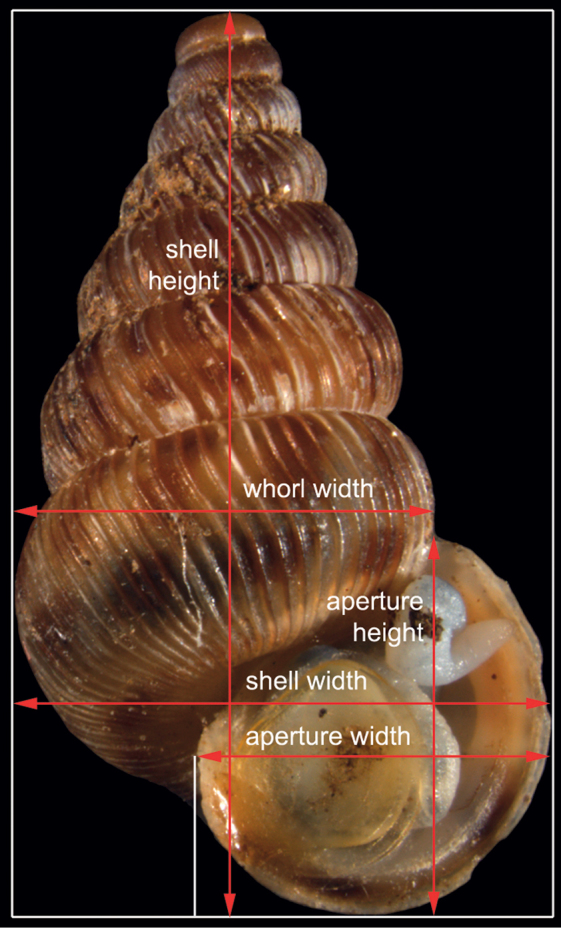
The 5 distances measured on the shell of *Cochlostoma septemspirale*: shell height and width, aperture height and width, and whorl width.

In their papers on the systematics of Pyrenean and Cantabrian cochlostomatid species, Raven (1990: 21 Fig. 1) as well as Gofas (2001) already mentioned that there is a sexual shell dimorphism which is reflected in the shells of males being smaller and stouter than females. According to Maassen (2007), this phenomenon is not uncommon among operculated families. In contrast, AnimalBase without referring the source states that there is no significant sexual dimorphism in *Cochlostoma septemspirale* (last visit July 2011). As the shell is one of the most useful characters for species recognition in *Cochlostoma* (Gofas 2001) it might also be useful for identification of the sex. As far as known, sex dimorphism in this species is not expressed by differing behaviour between the sexes. However, increasing fecundity with increasing body size in females could be a good reason for sex dimorphism (Shine 1989). There are some differences in the reproductive system that might lead to sexual dimorphisms in shells. Females have to produce eggs, so it would be logic that they need a larger body, resulting in a higher volume of the shell which should be measurable in some shell metric Gofas (2001) mentioned a difference in pigmentation of the shell between the sexes in some *Cochlostoma* species, but gives no explanation for this difference. A last feature that often plays an important role in taxonomic studies is the rib density (ribs/mm) on the whorls of the shell (Welter-Schultes 2010). It is known that this feature is usually quite homogeneous within a single population, but it also can show an important geographical variation (Gofas 2001). For this reason, a comparison of this feature between the sexes is interesting as well.

In this study, *Cochlostoma septemspirale* (Razoumowsky, 1789) is used to find such sex-related shell features. The distribution of *Cochlostoma septemspirale* extends from the Pyrenees to southern Germany and the central Balkans (Wagner 1897). In Switzerland, *Cochlostoma septemspirale* is especially common throughout the Jura, from where it has been described by Razoumowsky in 1789. Although *Cochlostoma septemspirale* is one of the most widely distributed *Cochlostoma* species, there is not much known about ecology, physiology, and reproduction biology of this species to date.

## Material and methods

For analysing differences between males and females, only mature individuals have been tested. These can be recognised by a thickened outer lip (Gofas 2001).

The aim of this study was to analyse different shell characteristics of 5 populations with 30–50 individuals per population. The seven main characters were (1) height and (2) width of the shell, (3) height and (4) width of the aperture, (5) width of the last whorl, (6) rib density on the last whorl, and (7) intensity of the reddish or brown pigments forming three bands over the shell (for definition of measurements, see Fig. 1).

### Field procedures

The collection of the 220 samples of *Cochlostoma septemspirale* was performed during two days in the Jura. Here, 6 sites had been sampled; the collection time on each site was about an hour. All collected specimens were preserved in plastic bags and later processed in the lab.

### Localities

Snails have been collected on the 5^th^ and on the 19^th^ of April. The first site was situated above Le Landeron (NE) (6 individuals); the second site was above La Neuveville (BE) (42 individuals), and the third site near Orvin (BE) (35 individuals) On the19^th^ of April, the first two sites were situated near Nods (BE) with 35 and 47 individuals respectively; the last site was on the Twannberg (BE) (55 individuals). The habitat structure of these sites was similar: south-facing slopes, mainly beech forest, with some interspersed little limestone boulders; usually with low shrubs and a herb layer and also a moderately thick layer of foliage. For more information to the localities refer to Appendix 1.

### Separation of the sexes

To sex the collected specimens, it is necessary to check the genital organs of each specimen. This cannot be done in the contracted state, when the animal is retracted in the shell with the operculum closing the aperture. To relax the animals, they were stored in bottles filled with water over night. In a next step, the relaxed specimens were poured with boiling water to fix them in the expanded state. After this procedure, males and females could easily be separated with the naked eye even. Only in three cases, the stereo-microscope was needed. All individuals were stored in alcohol (85%) until the next step.

### Measurements and conservation

Each specimen was photographed in a standardized orientation in up to 10 subsequent steps using a microscope camera (Leica DFC425). Afterwards, these multifocus images where combined to a single image by using ImageAccess Standard software. The measuring module of this software provided then measurements of height and width of shell, height and width of aperture and the width of the last whorl. The numbers of ribs on the last whorl and the pigmentation were also examined on the photos. Each specimen was stored in a glass tube with an individual number and preserved in 85% alcohol. Each lot received a catalogue number and was stored in the collection of the NMBE providing individual access to each specimen. All photos are stored on the NMBE Server and are available for subsequent research.

### Data analysis

Continuous characters (measurements): 5 continuous variables (Table 1) were treated with a multivariate analysis in order to explore the most significant variation in size and shape of the shell. According to Jolicoeur and Mosimann (1960), shape in general tends to provide more reliable information than size on the morphology of organisms. Size is often considered as a nuisance because it is strongly dependent on ecological factors (e.g., McCoy et al. 2006). Separation of size and shape in multivariate studies of morphological data is problematic (e.g., Claude 2008). One way to address this problem is by using principal component analysis (PCA), where the first principal component of PCA is usually considered as a general size axis, while the remaining principal components represent the shape space. However, the first principal component also includes size related shape information (Jolicoeur and Mosimann 1960) and has been identified by Jolicoeur (1963) heuristically as a multivariate allometric size axis (see Baur and Leuenberger 2011 for a model based statistical derivation). The mixture of size and size related shape information in the first component makes the interpretation of the other components of a PCA rather difficult. Baur and Leuenberger (2011) therefore have developed new methods that allow the interpretation of principal components in terms of ratios. With their tools it is possible to strictly separated size and shape. In a first step, an isometric size axis (below called “isosize”) is defined as the geometric mean of the original measurements and thus comprises only differences in scaling (for the exact definition isosize, see Baur and Leuenberger (2011: 816)). Isometry free shape variables are obtained by projecting the measurements orthogonal to isosize. A PCA calculated on the covariance matrix of these shape variables then accounts solely for differences in proportions. Baur and Leuenberger (2011) developed a graphical tool for the interpretation of principal components in shape space that they called the “PCA ratio spectrum”. To assess the amount of allometry in the data, Baur and Leuenberger (2011) suggested to plot the isosize against each significant shape component and introduced another graphical tool, the “allometry ratio spectrum”.

**Table 1.** Range, interquartile range, median, and number of specimens of each sex for the 5 distance measurements. Significance levels of t-test: ‘***’ p < 0.001, ‘**’ p < 0.01, ‘*’ p < 0.05, ‘.’ p < 0.1, ‘ ’ p < 1. All measurements in μm.

shell height ***						
sex	min.	1st quartile	median	3rd quartile	max.	N
females	6915	7428	7572	7780	8261	104
males	6549	6956	7120	7271	7873	116
**shell width**						
sex	min.	1st quartile	median	3rd quartile	max.	N
females	3615	3822	3878	3954	4207	104
males	3573	3837	3929	4003	4332	116
aperture height ***						
sex	min.	1st quartile	median	3rd quartile	max.	N
females	2249	2535	2596	2681	2953	104
males	2189	2587	2689	2772	2962	116
**aperture width**						
sex	min.	1st quartile	median	3rd quartile	max.	N
females	2166	2504	2564	2652	2902	104
males	2161	2456	2564	2650	2893	116
whorl width ***						
sex	min.	1st quartile	median	3rd quartile	max.	N
females	3069	3202	3258	3325	3531	104
males	2994	3117	3175	3228	3383	116

To examine how well the sexes are separated, the data were subjected to a linear discriminant analysis (LDA). The performance of the LDA was assessed by means of cross validation (Rencher 2002: 310), where one specimen is omitted from the analysis and classified according to the discriminant function found for the remaining specimens in the data set. The number of correctly classified cases is a measure of how well the result will generalize for an independent data set. Finally, the “LDA ratio extractor” (see Baur and Leuenberger 2011) was applied for finding the best ratios for separating the sexes. The measure δ, also introduced by Baur and Leuenberger (2011: 818, formula 14), was calculated to see how much of the total differences are due to size and how much are due to shape.

### Other characters

The rib density (ribs/mm) on the last whorl was determined on the same photographs that were used for taking measurements. For counting the ribs, the last whorl was magnified on the screen. The total number of ribs on the last whorl was divided by whorl width in mm.

Pigmentation has been recorded at the nominal level. There was no intention to include it in the PCA, because PCA has only been used for a size and shape analysis. To determine the intensity of the reddish or brown pigments, that form three bands over the shell, we also used the photographs of the specimens. The pigmentation was determined on the last whorl of the shell. The intensity of the bands was difficult to classify, so first the senior author worked out an appropriate method for classifying the intensity of pigmentation. Finally, a 3-level classification scheme (0 = missing band, 1 = interrupted band, and 2 = continuous band) was adopted and the shells were classified in a first run by the senior author. To avoid any bias, that easily might be introduced by a person involved in the study, an independent person repeated the classification of specimens in a second run.

All statistical and graphical analyses were performed with R, version 2.11.1 (R Development Core Team 2010). For the calculation of the multivariate methods of Baur and Leuenberger (2011) slightly modified versions of the R-scripts provided by the authors were employed (see section Supplementary material of their paper). In order to decide how many components to retain we plotted the scree graph (Rencher 2002: 398–399). Fisher’s exact test was applied for contingency tables (frequencies of pigmentation categories) and therefore the function “fisher.test()” was used. For testing differences in the mean we applied the t-test by using the function “t.test()” or the non-parametric Wilcoxon rank sum test and the function “wilcox.test()”, in case the distribution of the data points deviated from normality. The fit to a normal distribution was checked with Quantile-Quantile plots, i.e. by plotting the respective variables with functions “qqnorm()” and “qqline()”.

### Data resources

The data underpinning the analyses reported in this paper are deposited in the Dryad Data Repository at doi: http://dx.doi.org/10.5061/dryad.ns7v7.

## Results

### Size and shape analysis of distance measurements:

To get an overview, the range, interquartile range and median of the 5 continuous shell characters are given in Table 1. The shells of females are higher and the last whorl is wider compared to males, whereas the aperture is higher in males (t-tests, p < 0.001). For the two remaining characters, no significant differences between sexes were found.

The multivariate analysis offered a closer look concerning the variation in size and shape of the shells of *Cochlostoma septemspirale*. As mentioned in the Material and methods section, we calculated isosize (i.e., the geometric mean of all variables) as a general size measure. Although the range was more or less overlapping, mean isosize was significantly higher for females compared to males (Wilcoxon test, p < 0.01).

**Figure 2.**
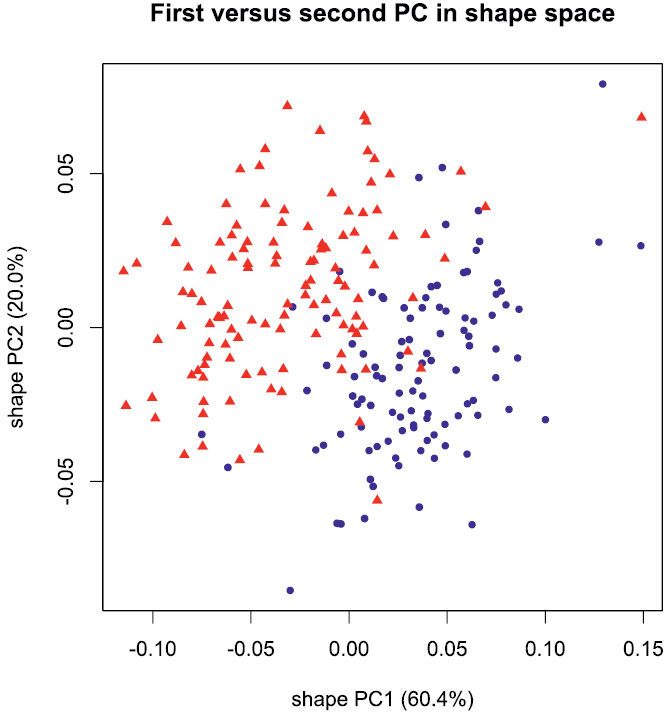
Scatterplot of first against second principal component in shape space; blue dots females, red triangles males.

**Figure 3.**
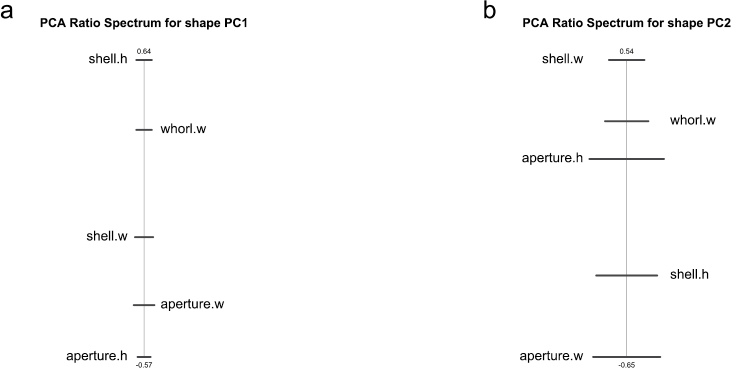
PCA ratio spectrum of first (**a**) and second principal component (**b**) in shape space.

A principal component analysis (PCA) performed in isometry free shape space allowed us to explore variation in shape. The scree graph (not shown) suggested the first two principal components (PC) which explained 80.4% of the total variance in the data. In Figure 2, the scatterplot of shape PC1 against shape PC2 is presented. In the graph, a distinct shift in the shape of females and males is evident, as the two cluster of points are only moderately overlapping. The shift is rather pronounced along shape PC1, but only weakly discernible along shape PC2. For the interpretation of the two components we used the PCA ratio spectrum (Fig. 3) as detailed by Baur and Leuenberger (2011). Following their description, ratios calculated from characters lying far apart in the spectrum explain a large portion of the variance of a shape PC; on the other hand, ratios from close characters contribute very little. According to the PCA ratio spectrum in Fig. 3a, shape PC1 was dominated by the ratio shell height:aperture height, while the PCA ratio spectrum of shape PC2 (Fig. 3b) suggested the ratio shell width:aperture width.

**Figure 4.**
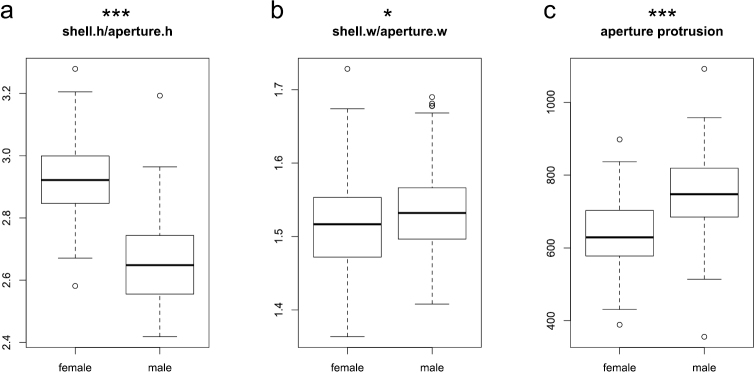
Boxplots of the ratios shell height:aperture height (**a**) and shell width:aperture width (**b**), and aperture protrusion (**c**). Significance levels of Wilcoxon test: ‘***’ p < 0.001, ‘**’ p < 0.01, ‘*’ p < 0.05, ‘.’ p < 0.1, ‘ ’ p < 1.

The first ratio, shell height:aperture height, showed a large mean difference between sexes (Wilcoxon test, p < 0.001; Fig. 4a). Females have a larger shell height as well as a smaller aperture height than males (Table 1). Because the width of the last whorl is also larger in females (Table 1), their shells should have considerably more volume in the part above the aperture. Indeed, mean volume is 1.19 times larger for females.

The mean of the other ratio, shell width:aperture width, differed slightly between sexes (Fig. 4b), although the differences in the means of shell width and aperture width are themselves not significant (Table 1). Mean shell width is nevertheless slightly larger in males, which is why the differences in the ratio became slightly significant (Wilcoxon test, p = 0.044). Because shell width and aperture width do not differ between sexes, the greater whorl width should therefore cause a less pronounced lateral protrusion of the female aperture. Indeed, aperture protrusion – calculated by subtracting whorl width from shell width – has distinctly lower values for females than for males (Wilcoxon test, p < 0.001; Fig. 4c).

**Figure 5.**
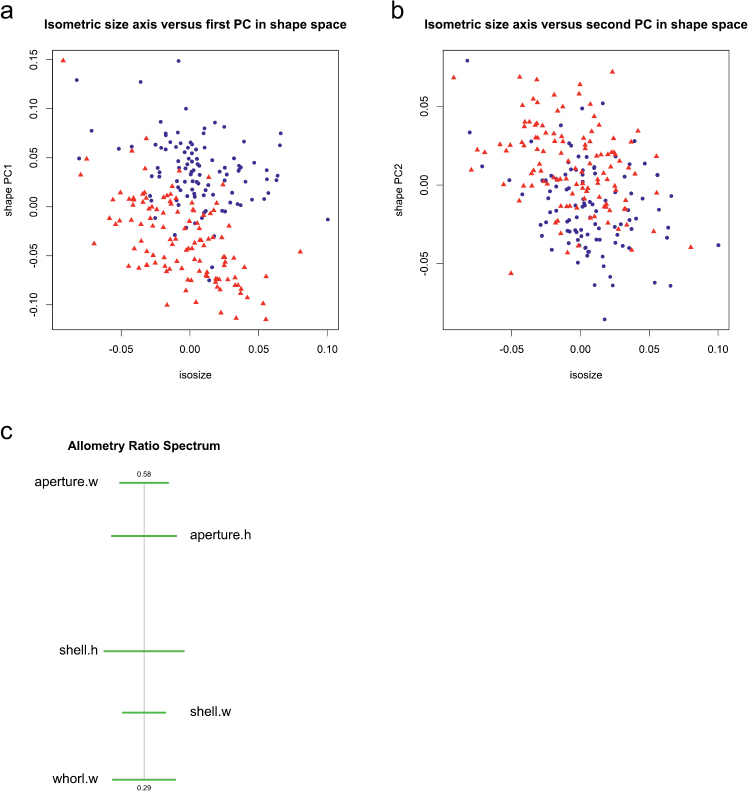
Scatterplots of isosize against first (**a**) and isosize against second principal component (**b**) in shape space; blue dots females, red triangles males. Allometry ratio spectrum (**c**).

To assess the amount of allometry in the data we plotted isosize against each of the shape PCs, in order to see how strongly shape correlates with size. Judging from the respective graphs (Figs 5a and 5b) only a very moderate correlation of shape PC2 with isosize is discernible, shape PC1 is clearly uncorrelated. Therefore allometric variation was of marginal importance concerning our data set. This observation is further supported by the allometry ratio spectrum. The latter is used in a very similar manner as a PCA ratio spectrum, except that the ratios calculated from distant characters are those showing the most distinctive allometric behaviour. Inspection of the allometry ratio spectrum in Figure 5c reveals aperture width:whorl width as the ratio with the strongest allometric variation. Obviously, it is not among the most dominant ratios of the PCA ratio spectrum of shape PC1 or shape PC2 (Fig. 2).

Often, a researcher might simply wish to identify the sex of a shell. We therefore performed a linear discriminant analysis (LDA) that reveals the best separation of two groups by using the total information in the data. The performance of the LDA was then assessed with cross validation (Table 2). In our analysis, it was possible to correctly classify 92.3% of the females and 88.7% of the males.

**Table 2.** Identification of specimens based on cross validation.

**sex**	**identification as**		**correctly identified**
	**female**	**male**	
females	96	8	92.3%
males	13	103	88.8%

**Figure 6.**
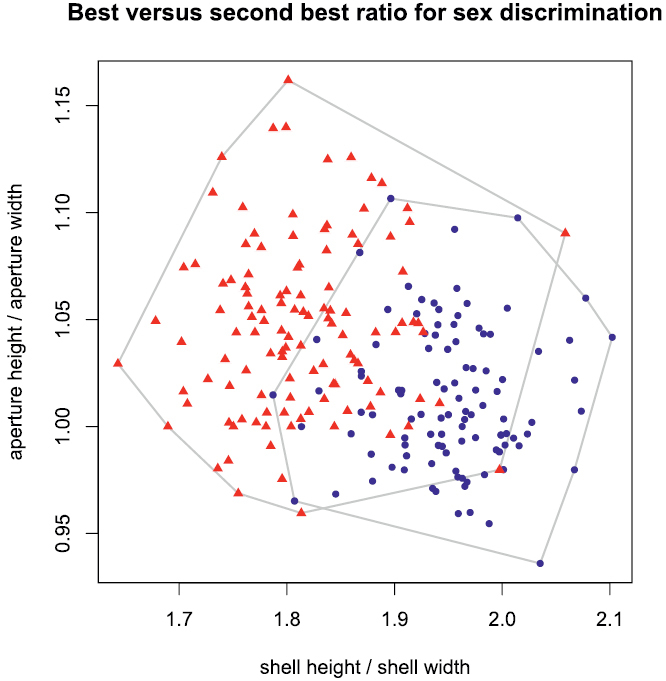
Scatterplot of best (shell height:shell width) against second best ratio (aperture height:aperture width) for separating females from males; blue dots females, red triangles males.

For practical reasons, a few characters that would allow quick and easy identification of most specimens might sometimes be useful, for instance in field work. A discriminant function is too awkward for such purpose, because its application is complicated and it lacks an intuitive element. One or two ratios would be preferable, as these are easily calculated and differences in proportions can sometimes even be estimated by eye. We therefore applied the LDA ratio extractor (see Baur and Leuenberger 2011: 816–817) for finding the optimal ratios for separating females and males. The two best ratios were shell height:width and aperture height:width (Fig. 6). Unfortunately, the sexes overlapped quite considerably in their range which is why ratios are apparently of limited use for identification. Baur and Leuenberger (2011) also introduced the measure *δ* that indicates how well shape discriminates in relation to size. A value of delta close to unity means that separation is mainly due to size, whereas for a value close to zero mostly shape is important. In our case, *δ* is 0.139 for the first ratio vector and thus the separation mainly stems from variation in shape.

### Anaylsis of other characters

**Figure 7.**
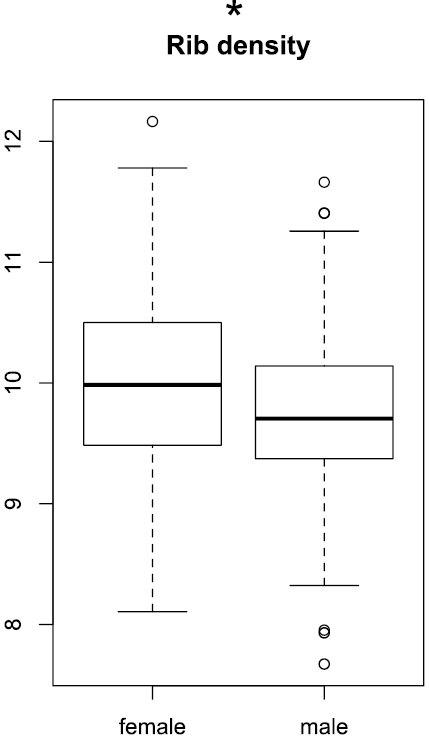
Boxplots of rip density. Significance levels of Wilcoxon test: ‘***’ p < 0.001, ‘**’ p < 0.01, ‘*’ p < 0.05, ‘.’ p < 0.1, ‘ ’ p < 1.

The rib density, determined on the last whorl, was largely overlapping between the sexes, although the mean was slightly higher for females (Wilcoxon test, p < 0.05; Fig. 7).

**Figure 8.**
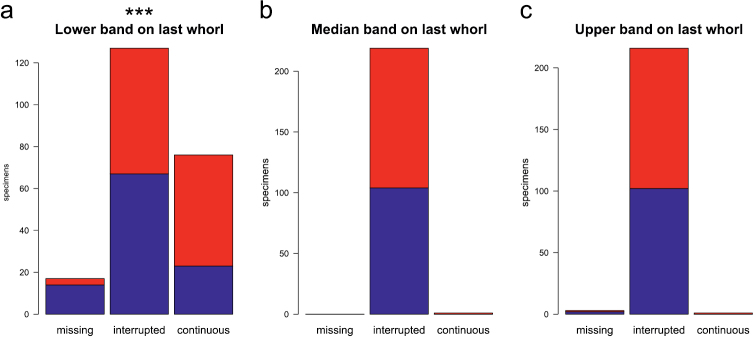
Bar diagrams showing the extent of pigmentation on the lower whorl: classification (missing, interrupted, continuous) of lower (**a**), median (**b**), and upper band (**c**); blue bars females, red bars males. Significance levels of Fisher’s exact test: ‘***’ p < 0.001, ‘**’ p < 0.01, ‘*’ p < 0.05, ‘.’ p < 0.1, ‘ ’ p < 1.

Pigmentation of the last whorl revealed a significant difference in the lower band, while pigmentation of the median and upper band was the same for males and females (Fig. 8). The figure is based on the classification made by the person that was not further involved in the study. However, classification by the senior author gave a very similar result.

## Sex ratio in the investigated six populations

The numbers of males and females within the investigated populations are presented in Table 3. Population no. 1 (n = 6) is excluded here, the sample size is considered to be too small. For the remaining five populations, the observed average of females/males approaches a value of nearly 1, i.e. there is an equal number of females and males present. Two populations show a value > 1 (= more females), two show a value < 1 (= less females), and one population is balanced.

**Table 3.** Sex ratio in five populations of *Cochlostoma septemspirale*.

**population**	**♀**	**♂**	**No. of specimens**	**sex ratio ♀/♂**
2	21	21	42	1.00
3	13	22	35	0.59
4	19	16	35	1.19
5	26	21	47	1.24
6	23	32	55	0.72
Sums	102	112	214	
mean				0.95

## Discussion

As expected, there is a sexual dimorphism in shells of *Cochlostoma septemspirale*. Females are larger than males, as revealed by larger mean isosize, a result already indicated by Gofas (2001). However, the main differences are due to a considerable variation in shell shape. Aperture height is reduced in females, while shell height and whorl width are both larger. Females therefore are not only larger, they also have about one fifth more volume in the central part of the teleoconch above the aperture.

These differences in size and shape of the sexes may be explained by the differing morphology of the reproductive systems between females and males. Females have a seminal receptacle and a bursa copulatrix well separated from the oviduct (Prince 1967; Varga 1984; De Mattia et al. 2011). The ovarium, which is situated in whorls 3 and 4 contains up to 40 eggs, which increase in size during growth and are transferred via the oviduct to the uterus in the last whorl. Finally, clutch size in *Cochlostoma septemspirale* reaches up to 10 eggs, with a diameter of 1.0–1.1 mm (Prince 1967). In contrast, males have only a germinative gland with a simple duct that ends in the penis on the right side of the head.

Mean rib density is slightly higher in females, but the ranges are more or less overlapping (Fig. 7).

In the character state “banding of the last whorl”, males are more strongly pigmented on the lower band than females (Fig. 8). Currently there is no explanation for this result, as males and females live in mixed populations under the same environmental conditions. It also cannot play a role in sex recognition, because the optical abilities of snails are limited to recognition of darkness or brightness (Chase 2001).

From Figure 2 and 6 it is evident that continuous character variation is quite strongly overlapping between sexes. However, the result of the cross validation indicated that about 90% of the shells can be correctly assigned (Table 2).

From the results of the calculation of the sex ratios it can be concluded that the ratio between males and females is approximately balanced. However, the number of investigated populations is much too small to come to a generalized conclusion. The observed ratio variability can also be due to a too small population sample.

As we have shown above, multivariate morphometric methods could be successfully applied to populations of *Cochlostoma septemspirale* from the Jura. In particular, it was possible to uncover subtle, but nevertheless important morphological differences between sexes, that otherwise might have gone unnoticed. Future investigations will show whether our results apply for other populations from the distribution area of *Cochlostoma septemspirale* or even other species of *Cochlostoma*. In practice, the methods are easily applicable in a wide variety of ecological and systematic studies and offer the additional benefit to exploit museum’s material with their mainly dry shell collections.

## Appendix 1

**Localities**

NMBE30979

Le Landeron, 47.06760435°N, 7.06510484°E (DD), 596 m, 5.4.2011, leg. Reichenbach et al.

NMBE30980

Chasseral, at the crossroads Diesse / Nods Chasseral, 47.06996106°N, 7.09448308°E (DD), 620 m, 5.4.2011, leg. Reichenbach et al.

NMBE30981

Orvin, 47.15439027°N, 7.19399571°E (DD), 833 m, 5.4.2011, leg. Reichenbach et al.

NMBE30982

Route de Chasseral, Combes de Nods, 47.11286416°N, 7.04331994°E (DD), 1154 m, 19.4.2011, leg. Reichenbach et al.

NMBE30983

Route de Chasseral, 1 km towards Nods, 47.12018349°N, 7.07096815°E (DD), 1054 m, 19.4.2011, leg. Reichenbach et al.

NMBE30984

Gaicht in direction to Twannberg, 47.10991059°N, 7.15971977°E (DD), 794 m, 19.4.2011, leg. Reichenbach et al.
